# Immune Infiltration Subtypes Characterization and Identification of Prognosis-Related lncRNAs in Adenocarcinoma of the Esophagogastric Junction

**DOI:** 10.3389/fimmu.2021.651056

**Published:** 2021-05-28

**Authors:** Xin Hu, Liuxing Wu, Ben Liu, Kexin Chen

**Affiliations:** Department of Epidemiology and Biostatistics, National Clinical Research Center for Cancer, Key Laboratory of Molecular Cancer Epidemiology of Tianjin, Tianjin Medical University Cancer Institute and Hospital, Tianjin, China

**Keywords:** adenocarcinoma of the esophagogastric junction, lncRNA, prognosis, TCGA, immune cell infiltration, immune risk signature

## Abstract

The incidence of adenocarcinoma of the esophagogastric junction (AEG) has markedly increased worldwide. However, the precise etiology of AEG is still unclear, and the therapeutic options thus remain limited. Growing evidence has implicated long non-coding RNAs (lncRNAs) in cancer immunomodulation. This study aimed to examine the tumor immune infiltration status and assess the prognostic value of immune-related lncRNAs in AEG. Using the ESTIMATE method and single-sample GSEA, we first evaluated the infiltration level of 28 immune cell types in AEG samples obtained from the TCGA dataset (N=201). Patients were assigned into high- and low-immune infiltration subtypes based on the immune cell infiltration’s enrichment score. GSEA and mutation pattern analysis revealed that these two immune infiltration subtypes had distinct phenotypes. We identified 1470 differentially expressed lncRNAs in two immune infiltration subtypes. From these differentially expressed lncRNAs, six prognosis-related lncRNAs were selected using the Cox regression analysis. Subsequently, an immune risk signature was constructed based on combining the values of the six prognosis-associated lncRNAs expression levels and multiple regression coefficients. To determine the risk model’s prognostic capability, we performed a series of survival analyses with Kaplan–Meier methods, Cox proportional hazards regression models, and the area under receiver operating characteristic (ROC) curve. The results indicated that the immune-related risk signature could be an independent prognostic factor with a significant predictive value in patients with AEG. Furthermore, the immune-related risk signature can effectively predict the response to immunotherapy and chemotherapy in AEG patients. In conclusion, the proposed immune-related lncRNA prognostic signature is reliable and has high survival predictive value for patients with AEG and is a promising potential biomarker for immunotherapy.

## Introduction

Adenocarcinoma of the esophagogastric junction (AEG) refers to adenocarcinoma within 5cm of the esophagogastric junction (EGJ). The broadly accepted definition of AEG was proposed by Siewert et al. (1). AEG is one of the most typical causes of cancer mortality worldwide and remains a challenging issue in oncology. Although AEG is less common than squamous cell carcinoma, for reasons unknown, the frequency of AEG has dramatically increased annually in both Western and Eastern countries over the last three decades for uncertain reasons (2). Emerging therapeutic strategies such as immunotherapy and targeted therapy have brought hope for patients with cancers such as gastrointestinal tumors (3). However, the response to existing immune-based treatments varies among individuals.

Modulation of diverse cells in the digestive tract tumor microenvironment (TME) influences tumorigenesis, and immunosuppressive microenvironments are associated with digestive tract tumor progression and poor prognosis (4, 5). Moreover, immunosuppressive TME remains a major obstacle for effective cancer immunotherapy. There are numerous invading immune cells in cancer tissues comprising T-cells, natural killer cells, and B-cells. In gastric cancer, NK infiltration is associated with better outcomes (6). In addition, studies have shown that the weakening of T-cell immune function after radiotherapy will affect the host’s immune response, which might be a critical factor affecting the prognosis of esophageal cancer (7). Strong implications between TME immune cells and cancer cells play a crucial role in tumorigenesis and cancer progression. Thus, enhancing immune cell function has emerged as an immunotherapy strategy in AEG. Therefore, to improve immune therapy response rates, there is a pressing need to provide a precisely screening program and get accurate and credible predictive biomarkers for efficacy of immunotherapy and prognostic of AEG patients.

LncRNAs are non-coding transcripts with >200 nucleotides. The mechanism underlying the function of lncRNA in cancer is very complicated. For instance, lncRNA H19 facilitates glioma angiogenesis *via* the miR-138/HIF-1α/VEGF cascade (8), while lncRNA TUC338 promotes invasion of lung cancer by activating MAPK signaling (9). LINC01094 was reported to promote carcinoma development in renal clear cell carcinoma and glioma cancer (10, 11). Another study on lncRNA gene cluster MIR100HG showed that two microRNAs (miR-100 and miR-125b) derived from this cluster can lead resistance to chemotherapeutic drugs through the Wnt pathway (12). A recent study further indicated that lncRNAs modulated immune function (13). The lncRNA NRON has been shown to maintain a resting state of T cells by sequestering phosphorylated NFAT in the cytoplasm (14). The oncogenic lncRNA LINK-A downregulates cancer cell antigen presentation and intrinsic tumor suppression (15). In addition, several studies have shown that immune-related lncRNA is a novel prognostic marker with prognostic value for cancer patients (16–18).

In the present study, we identified two immune infiltration subtypes of AEG based on 28 immune cell types and calculated the differently expressed lncRNAs between these two immune subtypes. Furthermore, we demonstrated the six immune-associated lncRNAs correlated with AEG prognosis and constructed an immune risk model using these six lncRNAs. Finally, we evaluated the predictive role of immune risk signature, both in immunotherapy and chemotherapy cohorts.

## Materials and Methods

### Collection and Grouping of AEG Data

This study used public data from the TCGA and UCSC Xena databases. According to Siewert classification, we included 201 histology confirmed AEG samples with complete survival information from stomach cancer(STAD) and esophageal cancer(ESCA) samples. Detailed patient characteristics of AEG are given in the **Supplementary Table S1**. The fragments per kilobase per million (FPKM) and counts data of the AEG RNA-seq were extracted from the TCGA program (https://portal.gdc.cancer.gov/). FPKM values were then transformed into transcript per million (TPM) values to estimate immune cells’ infiltration. Corresponding AEG clinical and mutation data were extracted from the UCSC Xena web data resource (https://xenabrowser.net/datapages/). Based on the lncRNA information in the GENECODE data resource V22 (https://www.gencodegenes.org/), we extracted the lncRNA expression profiles from the RNA-seq cohort. A set of biomarker genes for 28 types of immune cells was acquired from a past study (19). Next, we used ssGSEA to evaluate AEG infiltration by the 28 immune cells using the R package, GSVA (gene set variation analysis). Based on the ssGSEA results, AEG samples were clustered into the high (Immunity_H) or low immune cell infiltration (Immunity_L) groups using the R package, ConsensusClusterPlus. All resource, software, R packages, and protocols used herein are detailed in the key resource table and protocol workflow in **Supplemental Material**.

### Validation of the Effectiveness of Immune Subtypes

ESTIMATE R package was used to calculate Stromal Score, Immune Score, ESTIMATE Score, and Tumor Purity with TPM values of RNA-seq data. These analyses were used to evaluate the effect of ssGSEA grouping and draw a statistical map. Gene expression levels of various genes including members of the major histocompatibility complex (MHC), immune co-stimulator checkpoint (ICP), and immune co-inhibitor checkpoint (IAP) were also used to assess differences between two immune subtypes.

### Gene Sets Enrichment Analysis

We performed gene set enrichment analysis (GSEA) using “clusterProfiler” in R package to investigate the biological process difference between immune infiltration subtypes. The Kyoto Encyclopedia of Genes and Genomes (KEGG) and Gene Ontology (GO) results are exhibited by a GSEA plot.

### Significantly Mutated Genes Landscapes in AEG and Mutation Patterns in Two Immune Subtypes

We recognized the significantly mutated genes (SMG) with the GenVisR tool in R package. Mutation signature analysis of two immune subtypes was conducted using R package MutationalPatterns and Maftools. We extracted the mutational signature of AEG data and compared them with the mutation database (COSMIC V2) by using the cosine similarity method (https://cancer.sanger.ac.uk/cosmic/).

### Determination of Immune-Linked lncRNAs in AEG

The Bioconductor edgeR package was employed to calculate differential gene expression based on RNA-seq counts data of differential immune subtypes. Differentially expressed lncRNAs (DElncRNAs) called immune-related lncRNAs, were determined using the cut-off thresholds of P<0.05 and |log2 fold change| > 1. The selected lncRNAs were employed to construct a prognostic signature.

### Risk Assessment Model Construction and Survival Analysis

We used the entire AEG dataset (201/201, 100%) as the training set. Then, the whole dataset was randomly split into a validation dataset (140/201, 70%) and a test dataset (61/201, 30%). The immune-related lncRNAs were subject to univariate Cox regression assessment to identify those linked to AEG overall survival (OS). Only those lncRNAs that were statistically significant (P<0.05) were enrolled in multiple stepwise regression analysis. A risk assessment model for the patients was then developed using multivariate regression coefficients of lncRNA expression. Thus, we constituted the risk score by combining the expression value of included lncRNAs weighted by the linear regression model coefficients. Patient risk scores were calculated as previously described (20) and by using the following equation:

Riskscore=Exp1∗Coe1+Exp2∗Coe2+Exp3∗Coe3+…Expi∗Coei.

The risk scores of AEG patients were computed using the risk-assessment model. The patients were assigned to a high- and a low-risk group based on the cut-off values calculated using the survminer package in R. The Kaplan–Meier method was used to assess the efficiency of OS in high- and low-risk patients. The log-rank test was used to assess statistical significance at P<0.05.

### Chemotherapy and Immunotherapy Response With Immune-Linked lncRNAs Signature

The R package “pRRophetic” (21) was used to predict chemotherapeutic response in AEG patients. An immunotherapeutic data set of advanced urothelial cancer (IMvigor210 cohort) and a non-immunotherapeutic cohort of bladder cancer (BLCA) were used to validate the efficiency of immune risk signatures (22). Clinical information and gene expression data were extracted from the IMvigor210 data set (http://research-pub.gene.com/IMvigor210CoreBiologies). The non-immunotherapy cohort of bladder cancer (BLCA) was obtained from TCGA.

### Statistical Analysis

All statistical analyses were carried out in R (version 4.0.0; https://www.r-project.org). Mean ± SD was used to describe continuous variables that were normally distributed. Median (range) was used for continuous variables in non-normal distribution. Categorical variables were described as counts as well as percentages. P<0.05 (two-tailed t-test) represented statistical significance.

## Result

### Development and Validation of AEG Immune Subtypes

**Figure 1:**
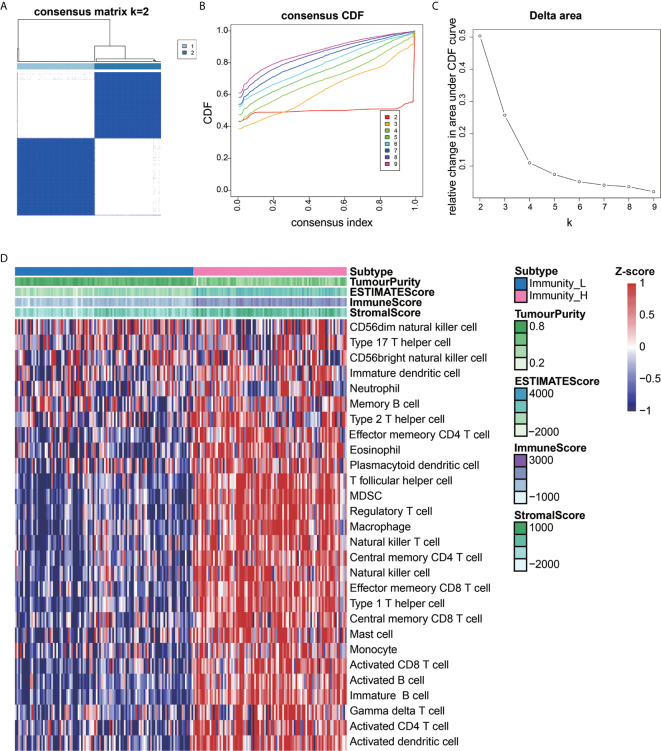
Construction of AEG immune infiltration. Single-sample gene set enrichment analysis (ssGSEA) identified the relative infiltration of 28 immune cell type subpopulations with different immune infiltration subtypes. The relative infiltration of each cell type was normalized into a Z-score. **(A–C)** The optimal number of clusters (K=2) was determined from cumulative distribution function (CDF) curves, and the classification effect is the best. **(D)** Patients with a low level of immune cell infiltration were named as the low immune cell infiltration subtype (Immunity_L), and those with a high level of immune cell infiltration were named as the high immune cell infiltration subtype (Immunity_H).

We extracted data from the TCGA and UCSC Xena resources. A total of 201 AEG samples accompanied with complete survival information were retained for our study. The consensus cluster analysis indicated that the optimal number of clusters was two, which was defined by CDF curves **(** **Figures 1A–C** **)**. According to the immune infiltration score, AEG samples were clustered into the high- and low- immune cell infiltration groups (N=93 and 108, respectively) **(** **Figure 1D** **)**.

**Figure 2:**
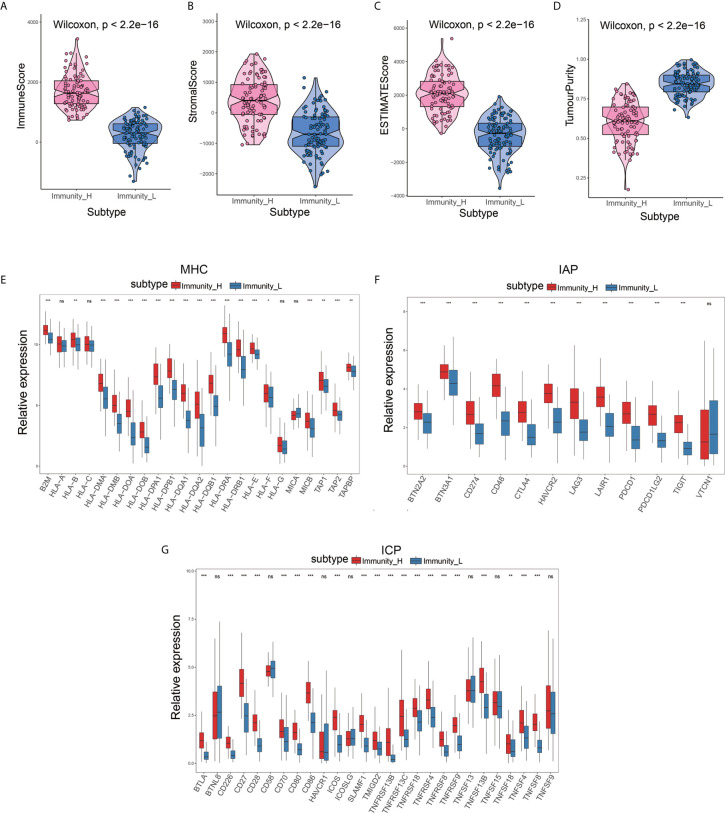
Validation of the effectiveness of immune subtypes. **(A–D)** The boxplot showed that there was a statistical difference in Immune Score, Stromal Score, ESTIMATE Score, and Tumor Purity between the two immune infiltration subtypes (P<2.2e-16). **(E–G)** The gene expression level of the gene set, including major histocompatibility complex (MHC), immune co-inhibitor checkpoints (IAP), and immune co-stimulator checkpoints (ICP) were all significantly different in the two immune infiltration subtypes (P<0.05).

To determine the feasibility of this grouping strategy, we used the ESTIMATE algorithm to compute Immune Score, Stromal Score, ESTIMATE Score, and Tumor Purity (P<2.2e-16) **(** **Figures 2A–C** **)**. The Boxplot analysis showed that there was a significant positive correlation between the high immune cell infiltration group (Immunity-H) and ESTIMATE Score, Immune Score, and Stromal Score, respectively. In contrast, there was a positive correlation only between the low immune cell infiltration group (Immunity-L) and Tumor Purity (P<2.2e-16) **(** **Figure 2D** **)**. Furthermore, we found that MHC, IAP, and ICP expression in the two immune cell infiltration groups were different (P<0.05) **(** **Figures 2E–G** **)**.

### Functional Annotation Related to the Two Immune Subtypes

**Figure 3:**
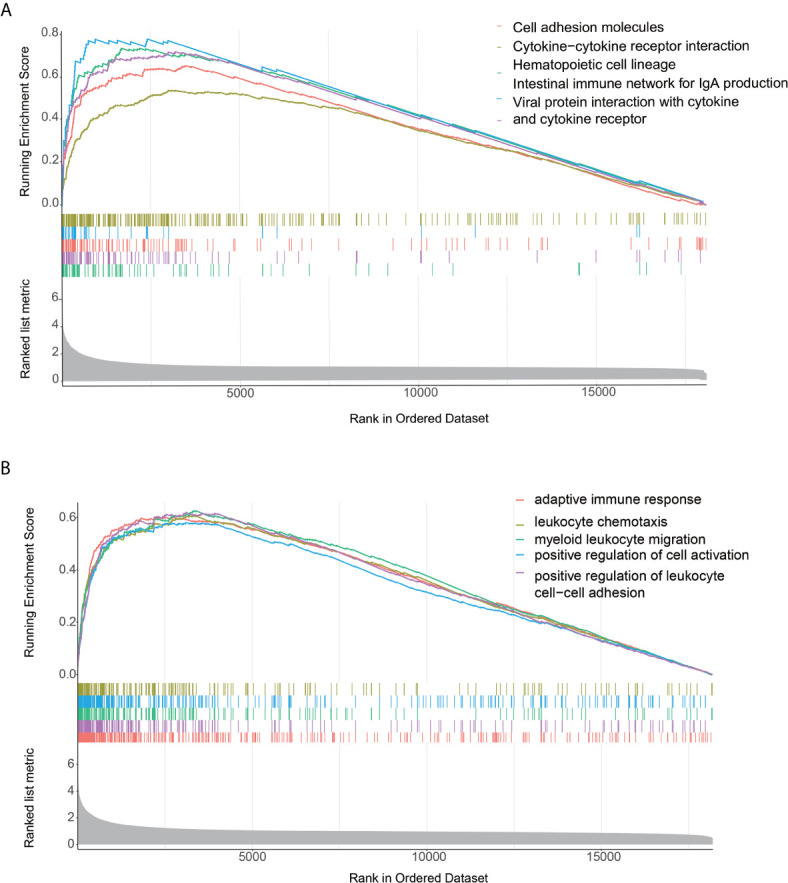
Functional annotation of the two immune infiltration subtypes. **(A, B)** Top enriched gene pathways/functions in distinct immune risk signature groups from the AEG cohort were assessed by using the GSEA algorithm.

The GSEA enrichment analysis demonstrated that many of these pathways are linked to the immune response in carcinoma **(** **Figures 3A, B** **)**. With Padjust<0.05 as the cut-off threshold, GO term enrichment analysis revealed that the genes were abundant in various processes, including adaptive immune response, positive modulation of cell activation, and positive modulation of leukocyte cell-cell adhesion. The KEGG pathway analysis indicated that these genes participated in cell adhesion molecules, cytokine-cytokine receptor cross-talk, and intestinal immune network for IgA production. The detailed GSEA results of two immune subtypes are provided in **Supplementary Table S2**.

### Analysis of Mutation Pattern Between High- and Low- Immune Cell Infiltration Groups

**Figure 4:**
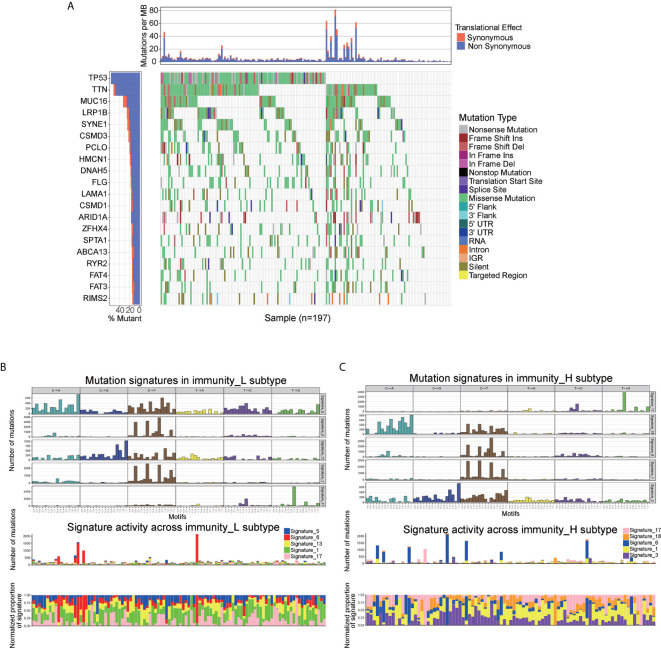
Mutational landscape of SMGs **(A)** in the TCGA AEG cohort. Mutation patterns **(B, C)** in the two immune infiltration subtypes.

To explore the association between immune cell infiltration and mutation pattern, we performed SMG analysis for AEG samples. The SMG mutational landscapes of AEG sample showed a distinct mutation ratio in TP53 (112/197 [56.6%]), TTN (102/197 [51.8%]), MUC16 (65/197 [33.0%]), and LRP1B (50/197 [25.4%]) **(** **Figure 4A** **)**.

To gain further insights into the operative mutational processes in two immune infiltration subtypes, we performed SMC and extracted the mutational signatures from the COSMIC database by using genomic somatic mutation data of AEG **(** **Figures 4B, C** **)**. The result revealed that immunity_L had the independent characteristics of signature 5 and signature 13, while immunity_H had the independent characteristics of signature 3 and signature 18. These results also showed that the mutation pattern of immunity_H was associated with DNA damage and repair pathways such as failed DNA double-strand break-repair through homologous recombination and AID/APOBEC pathway activity.

### Analysis of DElncRNAs between high- and low- immune cell infiltration subtypes

**Figure 5:**
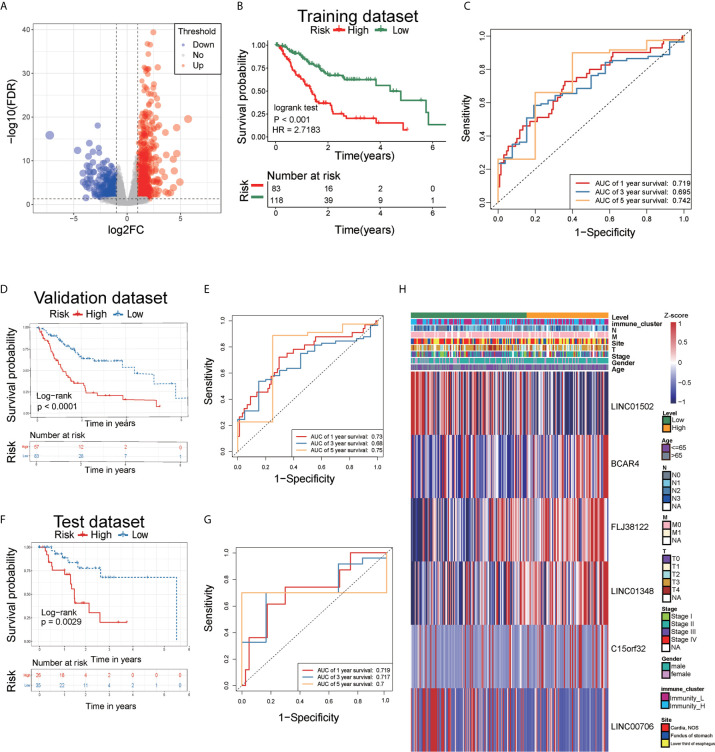
Analysis of differentially expressed lncRNAs and identification of immune-related lncRNA prognostic signature for AEG. **(A)** The volcano plot showed that 1016 lncRNAs were up-regulated and 454 down-regulated between the two immune infiltration subtypes. Each red dot showed an up-regulated lncRNA, and each blue dot shows a downregulated lncRNA (|Log2 Fold Chage| > 1 and FDR < 0.05). **(B)** The multiple stepwise regression analyses identified six lncRNAs correlated with prognostics. Patients in the high-risk group (red) exhibited worse overall survival (OS) than those in the low-risk group (green). **(C)** The receiver operator characteristic (ROC) curves to predict the sensitivity and specificity of 1-, 3-, and 5- years survival according to the 6-lncRNA signature-derived risk scores. **(D)** Kaplan–Meier analysis of the high versus low immune risk subgroup in validation dataset. **(E)** ROC curves to predict the sensitivity and specificity of 1-, 3-, and 5- years survival in validation dataset. **(F)** Kaplan–Meier analysis of the high versus low immune risk subgroup in test dataset. **(G)** ROC curves to predict the sensitivity and specificity of 1, 3, and 5 years survival in test dataset. **(H)** The expression of six lncRNAs in AEG patients.

We used the edgeR package to compare differential lncRNAs expression in high vs. low immune cell infiltration subtypes based on RNA-seq counts data. According to the cut-off thresholds of |Log2 Fold Change|>1 and FDR < 0.05, a total of 1470 lncRNAs that were differentially expressed were obtained, of which 1016 were upregulated and 454 were downregulated. **(** **Figure 5A** and **Supplementary Table S3** **).**

### Analysis of lncRNAs as Prognostic Biomarkers

A total of 1470 lncRNAs, which were differentially expressed, were analyzed *via* univariate Cox regression. Through univariate Cox proportional hazards regression analysis, 10 lncRNAs with prognostic significance (P<0.01) were identified. Using stepwise multiple regression analysis on these selected lncRNAs, we finally obtained six lncRNAs, namely LINC01502, FLJ38122, C15orf32, LINC00706, LINC01348, and BCAR4 **(** **Supplementary Table S4** **)**. Based on multiple stepwise regression analyses, a risk score was constructed as follows:

Riskscore=−0.2656×exp(LINC01502)+0.4971×exp(FLJ38122)+0.1952× exp(C15orf32)−0.2350×exp(LINC00706)+0.3437×exp(LINC01348)+0.4109×exp(BCAR4)

The cut-off value for the low-risk and high-risk groups was 0.1080, which was calculated by the R package survminer. Our data showed that the mortality rate of the high-risk group was markedly higher than that of the low-risk group **(** **Figure 5B** **)**, indicating that six lncRNAs played critical roles in AEG. The AUC for the 3- and 5-year survival was 0.695 and 0.742, respectively **(** **Figure 5C** **)**. We performed survival analyses (Kaplan-Meier test) on validation datasets and obtained similar results (P < 0.05) **(** **Figure 5D** **)**. The AUC for the 3- and 5-year survival was 0.73 and 0.75, respectively in validation datasets **(** **Figure 5E** **)**. Moreover, we performed survival analyses (Kaplan-Meier test) on test datasets and obtained similar results (P < 0.05) **(** **Figure 5F** **)**. The AUC for the 3- and 5-year survival was 0.719 and 0.7, respectively in test datasets **(** **Figure 5G** **)**. Heatmap analysis was used to visualize the expression of the six lncRNAs in AEG patient samples **(** **Figure 5H** **)**.

### Assessment of 6 Immune-Linked lncRNAs as Independent AEG Prognostic Factors

**Figure 6:**
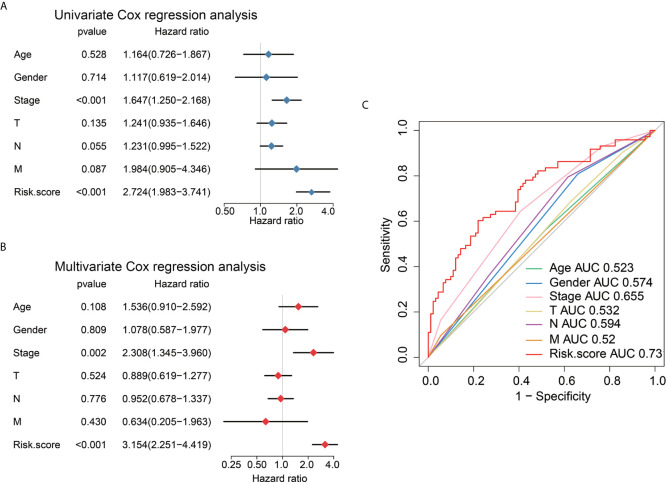
Univariate and multivariate analysis shows the prognostic value of 6-lncRNA signature. Univariate **(A)** and multivariate **(B)** Cox regression analyses of the association between clinicopathological factors and OS of AEG patients. **(C)** The receiver operator characteristic (ROC) curves to predict the sensitivity and specificity of clinicopathological factors and 6-lncRNA signature-derived risk scores in AEG patients.

Univariate Cox regression, multivariate Cox regression, and ROC analysis were employed to determine whether the six immune-related lncRNAs have prognostic value in AEG cancer independently of clinicopathological indicators such as age, pathological stage, and sex. The hazard ratio (HR) of risk score and 95%CI were 2.724 and 1.983–3.741 in the univariate Cox regression assessment (*P*<0.001), and 3.154 and 2.251–4.419 in the multivariate Cox regression assessment (*P*<0.001), respectively **(** **Figures 6A, B** **)**. Compared with the classic risk factor for pathological stage (AUC=0.655), the risk score (AUC=0.731) showed a better predictive power for survival in the TCGA AEG cohort (AUC=0.731) **(** **Figure 6C** **)**, which suggests that the six lncRNAs are independent AEG prognostic factors.

### 6-lncRNA Signature Can Predict the Response of Immunotherapy and Chemotherapy

**Figure 7:**
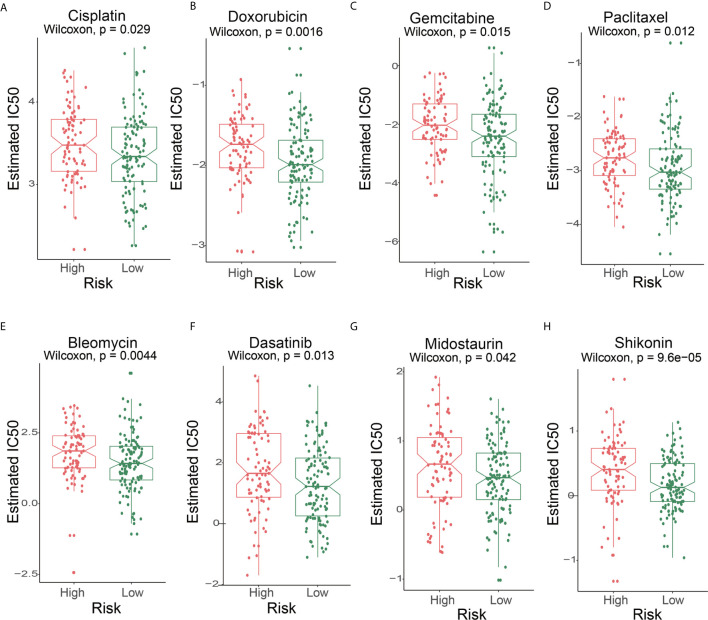
The IC50s of chemotherapeutic agents with 6-lncRNA signature. **(A)** cisplatin, **(B)** doxorubicin, **(C)** gemcitabine, **(D)** paclitaxel, **(E)** bleomycin, **(F)** dasatinib, **(G)** midostaurin, **(H)** shikonin.

First, we performed a prediction analysis of response to chemotherapy in the two risk groups by applying the “pRRophetic” method. Patients in the low-risk group had a lower estimated IC50 than those in the high-risk groups for the following chemotherapy drugs: bleomycin, cisplatin, dasatinib, doxorubicin, gemcitabine, midostaurin, shikonin, and paclitaxel **(** **Figures 7A-H** **)** (P<0.05).

**Figure 8:**
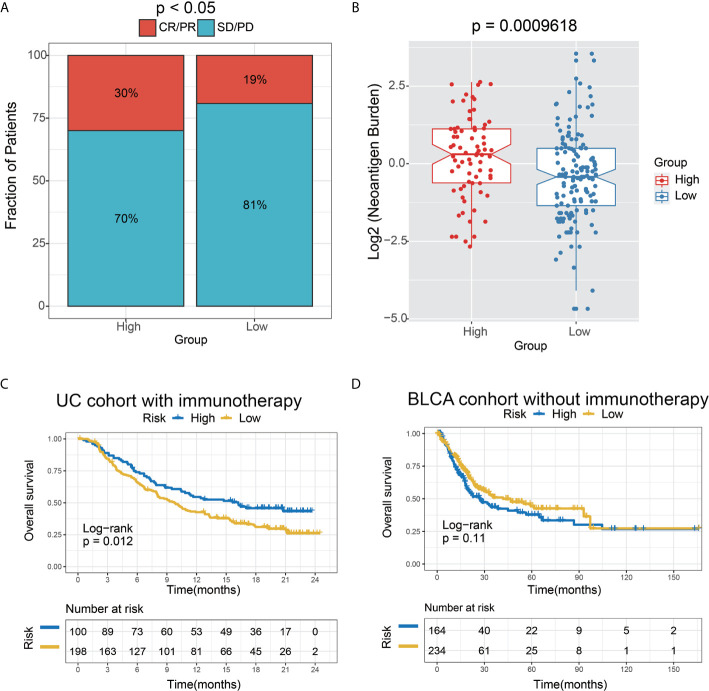
The 6-lncRNA signature in the role of immunotherapy. **(A)** The proportion of immune response to immunotherapy in high versus low immune risk score subgroups. CR, complete response; PR, partial response; SD, stable disease; PD, progressive disease. **(B)** Neoantigen burden in the UC cohort was compared among the distinct 6-lncRNA signature subgroups. **(C)** Kaplan–Meier analysis of the high versus low immune risk subgroup in the UC cohort. **(D)** Kaplan–Meier analysis of the high versus low immune risk subgroup in the BLCA cohort.

We further tested the predictor efficiency of the lncRNA risk model in the urothelial carcinoma (UC, the most common type of bladder cancer) cohort with immunotherapy (IMvigor210). The results on this validation set showed that the high-risk group had a higher immunotherapy response rate (P<0.05) and neoantigen burden (*P*=0.0009618) than the low-risk group, based on the 6-lncRNA signature **(** **Figures 8A, B** **)**. Interestingly, the Kaplan-Meier curves revealed that the high-risk group had improved survival than the low-risk group, contrary to the AEG non-immunotherapy cohort **(** **Figure 5B** **)**.

To verify the hypothesis that the prognosis of the high-risk group may be remarkably improved by immunotherapy, we employed the bladder cancer (BC) TCGA cohort as the control dataset based on the 6-lncRNA signature. Moreover, those BC patients who did not receive immunotherapy were selected for survival analysis. As expected, a trend toward unfavorable prognosis in the high-risk group was observed in the UC immunotherapy cohort (*P*=0.11) **(** **Figure 8D** **)**, which was opposite to the result in the UC immunotherapy cohort (*P*=0.012) **(** **Figure 8C** **)**.

## Discussion

Globally, AEG is the most common and fatal malignant tumor, with highly heterogeneous biological features (23). This study distinguished two novel immune subtypes in AEG samples based on the immune infiltration score. We observed apparent heterogeneity between two immune subtypes. It is known that the high heterogeneity of AEG exists not only in the genotypes and phenotypes of tumor cells but also in the TME (24). The TME is comprised of numerous cell types including cancer cells, immune cells, stromal cells, and fibroblasts. Therefore, this high complexity of immune cells may be the main reason for the heterogeneity in two immune infiltration subtypes. This finding was consistent with previous reports. Derks et al. (25) confirmed substantial heterogeneity in the TME between distinct subtypes in gastroesophageal adenocarcinomas. They also elucidated tertiary lymphoid structures(TLSs) in half of diffuse/genome-stable (GS) gastric cancers. It is worth noting that the subjects in Derks et al.’s study included those with gastric cancer and esophageal cancer, while our subjects had AEG.

Tumor immune cell infiltration is known to be associated with the outcome of gastroesophageal adenocarcinomas. For instance, Zhang et al. proved that high tumor-infiltrating lymphocytes (TIL) levels were associated with a favorable prognosis and that TIL reflected a protective host antitumor immune response (26). High levels of myeloid-derived suppressor cells (MDSCs) were associated with poor prognosis and therapeutic resistance in esophageal cancer (27, 28). However, there is a lack of such prognosis studies in AEG patients. In the current research, we found no significant difference in the overall survival of AEG patients between two immune infiltration groups **(** **Supplementary Figure S1** **)**. This result has been confirmed by Derks et al. (25).

Further investigation on the relationship between TME and prognosis in AEG may improve the outcome for AEG patients. In addition, interestingly, we found that the immune infiltration situation was different in male and female patients **(** **Supplementary Table S1** **)**. Female patients seemed to have a higher immune infiltration state in AEG. This result implied that sex-based differences should be considered for personalized antitumor immunotherapy in AEG patients. The study on detailed characteristics of tumor immune infiltration can provide personalized guidance and potential candidates for the immunotherapy of cancer patients. In the current study, we focused on the heterogeneity of AEG and the interaction between tumor-infiltrating immune cells and tumor cells, which was necessary to study the mechanism of tumor progression and develop new diagnostic and antitumor immune therapeutic approaches. Multiple genomic features such as tumor non-synonymous mutation load (TML) and mutational signatures have shown a strong correlation with clinical response to ICI treatment (29). To our knowledge, this is the first study on the tumor immune infiltration landscape in AEG samples. A key finding of the current study is that we revealed a different mutational signature profile between the two immune subtypes of AEG patients.

There is emerging evidence in the literature indicating that dysregulation of lncRNAs involved in the regulation of the immune system (30). Further, several lines of evidence identified these lncRNAs as immune-related lncRNA (31). For instance, Li et al. introduced an integrated algorithm, ImmLnc, which was employed to identify immune-related lncRNA. It is worth noting that even though the subjects in the above research spanned more than 30 cancer types, while AEG samples did not include in their study. Moreover, Li et al. performed a correlation analysis between the immune pathway and lncRNA to identify immune-related lncRNA in low-grade glioma (13). In addition, Shen et al. performed a similar approach as in our study to evaluated immune-related lncRNAs in breast cancer (32). Indeed, experimental supporting evidence is considered the gold standard for judging lncRNA function.

LncRNAs have been increasingly identified as a prognostic signature for cancer patients. For instance, Shen et al. evaluated prognosis using 11 immune-related lncRNAs in breast cancer (32). Numerous studies indicated that lncRNAs had been increasingly identified as prognostic signatures in cancer patients (33, 34). Using the transcriptome sequencing data and immune infiltration scores of AEG, we identified 6-lncRNA prognostic signatures related to immune cell infiltration in this study. The 6-lncRNA prognostic signature can predict the outcome and response to immunotherapy in AEG patients. BCAR4 is one member of the six lncRNAs. Recent studies have shown that BCAR4 can promote the migration and proliferation of tumor cells in various cancers (35–38). Notably, Godinho et al. found that BCAR4 related tamoxifen resistance in breast cancer patients (39). The functions of the other five lncRNAs, however, have not been reported so far. Further studies are warranted to assess the immunomodulatory role of BCAR4 and the other five lncRNAs in AEG.

The adenocarcinomas of gastroesophageal junction, either in the distal esophagus or gastric cardia, were considered to have a similar etiology. However, the adenocarcinomas arising in this site are heterogeneous and aggressive tumors with distinct malignant biological behaviors. We hypothesized that identifying the immune-related lncRNA risk model of AEGs might reveal novel molecular subgroups and may be beneficial to predict the prognosis and response to immunotherapy. There is growing evidence suggesting that AEG is a highly complex malignancy, comprising distinct subtypes associated with genetic and epigenetic alterations (24, 40, 41). For instance, the incidence of chromosomally unstable tumors was increased in gastro-esophageal junction adenocarcinomas (42). Our study addresses for the first time the features of lncRNA -related subgroup in AEG. Notably, our results did not converge well with the classical Siewert classification in AEG, which has implications for lymph node spread (43). Those results may explain apparently differ phenotypically or genetically between high- and low- risk groups.

Our study has some limitations. First is the modest sample size of AEG. We still need more AEG samples to verify the reliability of our conclusions. We still need more AEG samples to verify the reliability of our conclusions. Another pitfall is that the immune-based therapies data of AEG patients were not available now. More validation datasets of received immunotherapy are needed to verify the stability of immune-related lncRNA prognostic signature. Finally, the lncRNAs we mined have complications with the outcome of AEG patients, whereas the function of these novel non-coding RNAs is unclear. More experiments are desired to elucidate the underlying mechanism of these lncRNAs in tumor progression and immune escape.

In conclusion, we believe our findings highlight the critical implications of the tumor immune infiltration landscape and shed light on establishing a prediction model based on immune-related lncRNAs to predict the clinical outcome and immunotherapy responses.

## Data Availability Statement

Publicly available datasets were analyzed in this study. This data can be found here: https://portal.gdc.cancer.gov and https://xenabrowser.net/datapages/. For detail sample information see **Supplementary Table S5**. 

## Author Contributions

XH, BL, and LW contributed to writing, reviewing, and editing the article. XH and LW contributed to the development of methodology. BL and KC contributed to the administrative, technical, or material support. All authors contributed to the article and approved the submitted version.

## Funding

This work was supported by a grant from the National Natural Science Foundation of China (82073028, 81572445) to BL, National Key R&D Program of China (2017YFC0908300) to BL, and Natural Science Foundation of Tianjin (16JCYBJC24700) to BL and KC.

## Conflict of Interest

The authors declare that the research was conducted in the absence of any commercial or financial relationships that could be construed as a potential conflict of interest.

## References

[1] Deng JY Liang H. Adenocarcinoma of Esophagogastric Junction. Chin J Cancer Res (2014) 26(4):362–3. 10.3978/j.issn.1000-9604.2014.07.03 PMC4153928 25232205

[2] Rice TW Patil DT Blackstone EH. 8th Edition AJCC/UICC Staging of Cancers of the Esophagus and Esophagogastric Junction: Application to Clinical Practice. Ann Cardiothorac Surg (2017) 6(2):119–30. 10.21037/acs.2017.03.14 PMC5387145 28447000

[3] Kaufman HL Atkins MB Subedi P Wu J Chambers J Joseph Mattingly T 2nd. The Promise of Immuno-Oncology: Implications for Defining the Value of Cancer Treatment. J Immunother Cancer (2019) 7(1):129. 10.1186/s40425-019-0594-0 31101066 PMC6525438

[4] Leiting JL Grotz TE. Optimizing Outcomes for Patients With Gastric Cancer Peritoneal Carcinomatosis. World J Gastrointest Oncol (2018) 10(10):282–9. 10.4251/wjgo.v10.i10.282 PMC6198298 30364780

[5] Hong D Zhang X Li R Yu J Lou Y He Q. Deletion of TMEM268 Inhibits Growth of Gastric Cancer Cells by Downregulating the ITGB4 Signaling Pathway. Cell Death Differ (2019) 26(8):1453–66. 10.1038/s41418-018-0223-3 PMC6748091 30361615

[6] Ishigami S Natsugoe S Tokuda K Nakajo A Che X Iwashige H. Prognostic Value of Intratumoral Natural Killer Cells in Gastric Carcinoma. Cancer (2000) 88(3):577–83. 10.1002/(SICI)1097-0142(20000201)88:3<577::AID-CNCR13>3.0.CO;2-V 10649250

[7] Hong M Jiang Z Zhou YF. Effects of Thermotherapy on Th1/Th2 Cells in Esophageal Cancer Patients Treated With Radiotherapy. Asian Pac J Cancer Prev (2014) 15(5):2359–62. 10.7314/apjcp.2014.15.5.2359 24716984

[8] Liu ZZ Tian YF Wu H Ouyang SY Kuang WL. Lncrna H19 Promotes Glioma Angiogenesis Through Mir-138/HIF-1α/VEGF Axis. Neoplasma (2020) 67(1):111–8. 10.4149/neo_2019_190121N61 31777264

[9] Zhang YX Yuan J Gao ZM Zhang ZG. Lncrna TUC338 Promotes Invasion of Lung Cancer by Activating MAPK Pathway. Eur Rev Med Pharmacol Sci (2018) 22(2):443–9. 10.26355/eurrev_201801_14193 29424901

[10] Jiang Y Zhang H Li W Yan Y Yao X Gu W. Foxm1-Activated LINC01094 Promotes Clear Cell Renal Cell Carcinoma Development Via MicroRNA 224-5p/CHSY1. Mol Cell Biol (2020) 40(3):e00357-19. 10.1128/MCB.00357-19 31767633 PMC6965037

[11] Zhu B Liu W Liu H Xu Q Xu W. Linc01094 Down-Regulates miR-330-3p and Enhances the Expression of MSI1 to Promote the Progression of Glioma. Cancer Manag Res (2020) 12:6511–21. 10.2147/CMAR.S254630 PMC7395698 32801889

[12] Lu Y Zhao X Liu Q Li C Graves-Deal R Cao Z. Lncrna MIR100HG-Derived miR-100 and miR-125b Mediate Cetuximab Resistance Via Wnt/beta-catenin Signaling. Nat Med (2017) 23(11):1331–41. 10.1038/nm.4424 PMC5961502 29035371

[13] Li Y Jiang T Zhou W Li J Li X Wang Q. Pan-Cancer Characterization of Immune-Related lncRNAs Identifies Potential Oncogenic Biomarkers. Nat Commun (2020) 11(1):1000. 10.1038/s41467-020-14802-2 32081859 PMC7035327

[14] Sharma S Findlay GM Bandukwala HS Oberdoerffer S Baust B Li Z. Dephosphorylation of the Nuclear Factor of Activated T Cells (NFAT) Transcription Factor is Regulated by an RNA-protein Scaffold Complex. Proc Natl Acad Sci USA (2011) 108(28):11381–6. 10.1073/pnas.1019711108 PMC3136327 21709260

[15] Hu Q Ye Y Chan L-C Li Y Liang K Lin A. Oncogenic lncRNA Downregulates Cancer Cell Antigen Presentation and Intrinsic Tumor Suppression. Nat Immunol (2019) 20(7):835–51. 10.1038/s41590-019-0400-7 PMC6619502 31160797

[16] Liu Z Mi M Li X Zheng X Wu G Zhang L. Lncrna OSTN-AS1 may Represent a Novel Immune-Related Prognostic Marker for Triple-Negative Breast Cancer Based on Integrated Analysis of a ceRNA Network. Front Genet (2019) 10:850. 10.3389/fgene.2019.00850 31572452 PMC6753250

[17] Li JP Li R Liu X Huo C Liu TT Yao J. A Seven Immune-Related Lncrnas Model to Increase the Predicted Value of Lung Adenocarcinoma. Front Oncol (2020) 10:560779. 10.3389/fonc.2020.560779 33163400 PMC7591457

[18] Lin Y Pan X Chen Z Lin S Chen S. Identification of an Immune-Related Nine-LncRNA Signature Predictive of Overall Survival in Colon Cancer. Front Genet (2020) 11:318. 10.3389/fgene.2020.00318 32425969 PMC7203495

[19] Charoentong P Finotello F Angelova M Mayer C Efremova M Rieder D. Pan-Cancer Immunogenomic Analyses Reveal Genotype-Immunophenotype Relationships and Predictors of Response to Checkpoint Blockade. Cell Rep (2017) 18(1):248–62. 10.1016/j.celrep.2016.12.019 28052254

[20] Lin T Fu Y Zhang X Gu J Ma X Miao R. A Seven-Long Noncoding RNA Signature Predicts Overall Survival for Patients With Early Stage Non-Small Cell Lung Cancer. Aging (Albany NY) (2018) 10(9):2356–66. 10.18632/aging.101550 PMC6188476 30205363

[21] Geeleher P Cox N Huang RS. pRRophetic: An R Package for Prediction of Clinical Chemotherapeutic Response From Tumor Gene Expression Levels. PloS One (2014) 9(9):e107468. 10.1371/journal.pone.0107468 25229481 PMC4167990

[22] Mariathasan S Turley SJ Nickles D Castiglioni A Yuen K Wang Y. Tgfbeta Attenuates Tumour Response to PD-L1 Blockade by Contributing to Exclusion of T Cells. Nature (2018) 554(7693):544–8. 10.1038/nature25501 PMC6028240 29443960

[23] Siewert JR Feith M Stein HJ. Biologic and Clinical Variations of Adenocarcinoma At the Esophago-Gastric Junction: Relevance of a Topographic-Anatomic Subclassification. J Surg Oncol (2005) 90(3):139–46. 10.1002/jso.20218 15895452

[24] Bornschein J Wernisch L Secrier M Miremadi A Perner J MacRae S. Transcriptomic Profiling Reveals Three Molecular Phenotypes of Adenocarcinoma At the Gastroesophageal Junction. Int J Cancer (2019) 145(12):3389–401. 10.1002/ijc.32384 PMC6851674 31050820

[25] Derks S de Klerk LK Xu X Fleitas T Liu KX Liu Y. Characterizing Diversity in the Tumor-Immune Microenvironment of Distinct Subclasses of Gastroesophageal Adenocarcinomas. Ann Oncol (2020) 31(8):1011–20. 10.1016/j.annonc.2020.04.011 PMC7690253 32387455

[26] Zhang D He W Wu C Tan Y He Y Xu B. Scoring System for Tumor-Infiltrating Lymphocytes and Its Prognostic Value for Gastric Cancer. Front Immunol (2019) 10:71. 10.3389/fimmu.2019.00071 30761139 PMC6361780

[27] Chen M-F Kuan F-C Yen T-C Lu M-S Lin P-Y Chung Y-H. IL-6-Stimulated CD11b+ Cd14+ HLA-DR- Myeloid-Derived Suppressor Cells, are Associated With Progression and Poor Prognosis in Squamous Cell Carcinoma of the Esophagus. Oncotarget (2014) 5(18):8716–28. 10.18632/oncotarget.2368 PMC4226716 25238263

[28] Gabitass RF Annels NE Stocken DD Pandha HA Middleton GW. Elevated Myeloid-Derived Suppressor Cells in Pancreatic, Esophageal and Gastric Cancer are an Independent Prognostic Factor and are Associated With Significant Elevation of the Th2 Cytokine Interleukin-13. Cancer Immunol Immunother (2011) 60(10):1419–30. 10.1007/s00262-011-1028-0 PMC3176406 21644036

[29] Mandal R Samstein RM Lee K-W Havel JJ Wang H Krishna C. Genetic Diversity of Tumors With Mismatch Repair Deficiency Influences anti-PD-1 Immunotherapy Response. Science (2019) 364(6439):485–91. 10.1126/science.aau0447 PMC6685207 31048490

[30] Chen YG Satpathy AT Chang HY. Gene Regulation in the Immune System by Long Noncoding RNAs. Nat Immunol (2017) 18(9):962–72. 10.1038/ni.3771 PMC9830650 28829444

[31] Sun J Zhang Z Bao S Yan C Hou P Wu N. Identification of Tumor Immune Infiltration-Associated lncRNAs for Improving Prognosis and Immunotherapy Response of Patients With Non-Small Cell Lung Cancer. J Immunother Cancer (2020) 8(1):e000110. 10.1136/jitc-2019-000110 32041817 PMC7057423

[32] Shen Y Peng X Shen C. Identification and Validation of Immune-Related lncRNA Prognostic Signature for Breast Cancer. Genomics (2020) 112(3):2640–6. 10.1016/j.ygeno.2020.02.015 32087243

[33] Li J Wang W Xia P Wan L Zhang L Yu L. Identification of a five-lncRNA Signature for Predicting the Risk of Tumor Recurrence in Patients With Breast Cancer. Int J Cancer (2018) 143(9):2150–60. 10.1002/ijc.31573 PMC6519083 29707762

[34] Lian P Wang Q Zhao Y Chen C Sun X Li H. An Eight-Long non-Coding RNA Signature as a Candidate Prognostic Biomarker for Bladder Cancer. Aging (Albany NY) (2019) 11(17):6930–40. 10.18632/aging.102225 PMC6756879 31479417

[35] Zou R Chen X Jin X Li S Ou R Xue J. Up-Regulated BCAR4 Contributes to Proliferation and Migration of Cervical Cancer Cells. Surg Oncol (2018) 27(2):306–13. 10.1016/j.suronc.2018.05.013 29937186

[36] Yang H Yan L Sun K Sun X Zhang X Cai K. Lncrna BCAR4 Increases Viability, Invasion, and Migration of Non-Small Cell Lung Cancer Cells by Targeting Glioma-Associated Oncogene 2 (*GLI2*). Oncol Res (2019) 27(3):359–69. 10.3727/096504018X15220594629967 PMC7848411 29615150

[37] Ouyang S Zhou X Chen Z Wang M Zheng X Xie M. Lncrna BCAR4, Targeting to miR-665/STAT3 Signaling, Maintains Cancer Stem Cells Stemness and Promotes Tumorigenicity in Colorectal Cancer. Cancer Cell Int (2019) 19:72. 10.1186/s12935-019-0784-3 30962766 PMC6438025

[38] Chen F Mo J Zhang L. Long Noncoding RNA BCAR4 Promotes Osteosarcoma Progression Through Activating GLI2-dependent Gene Transcription. Tumour Biol (2016) 37(10):13403–12. 10.1007/s13277-016-5256-y 27460090

[39] Godinho MFE Sieuwerts AM Look MP Meijer D Foekens JA Dorssers LCJ. Relevance of BCAR4 in Tamoxifen Resistance and Tumour Aggressiveness of Human Breast Cancer. Br J Cancer (2010) 103(8):1284–91. 10.1038/sj.bjc.6605884 PMC2967058 20859285

[40] Geng Q Lao J Zuo X Chen S Bei JX Xu D. Identification of the Distinct Genomic Features in Gastroesophageal Junction Adenocarcinoma and its Siewert Subtypes. J Pathol (2020) 252(3):263–73. 10.1002/path.5516 32715475

[41] Lin Y Luo Y Sun Y Guo W Zhao X Xi Y. Genomic and Transcriptomic Alterations Associated With Drug Vulnerabilities and Prognosis in Adenocarcinoma At the Gastroesophageal Junction. Nat Commun (2020) 11(1):6091. 10.1038/s41467-020-19949-6 33257699 PMC7705019

[42] Van Cutsem E Sagaert X Topal B Haustermans K Prenen H. Gastric Cancer. Lancet (2016) 388(10060):2654–64. 10.1016/s0140-6736(16)30354-3 27156933

[43] Chevallay M Bollschweiler E Chandramohan SM Schmidt T Koch O Demanzoni G. Cancer of the Gastroesophageal Junction: A Diagnosis, Classification, and Management Review. Ann N Y Acad Sci (2018) 1434(1):132–8. 10.1111/nyas.13954 30138540

