# Supplementary material for: Immune Infiltration Subtypes Characterization and Identification of Prognosis-Related lncRNAs in Adenocarcinoma of the Esophagogastric Junction
Source: Front Immunol. 2021 May 28;12:651056. doi: 10.3389/fimmu.2021.651056 (PMC8195339; doi:10.3389/fimmu.2021.651056)
Supplement: Supplementary file 1 [file DataSheet_1.docx]

**Immune infiltration subtypes characterization and identification of prognosis-related lncRNAs in adenocarcinoma of the esophagogastric junction**

**Supplemental Information**

**Table S1**. The correlation between immune infiltration subtypes and clinical indexes in TCGA AEG cohort(*N* = 201)

**Table S2.** The results of gene sets enrichment analysis between two immune infiltration subtypes (*P* < 0.05)

**Table S3.** 1470 lncRNAs that were differentially expressed between two immune infiltration subtypes (FDR < 0.05 and | logFC | >1)

**Table S4.** Six prognostic risk signatures closely related to immune infiltration

**Table S5.** Requested public datasets of AEG samples in this study

**Table S6.** key resource table

**Figure S1.** Kaplan–Meier of the immunity_H versus immunity_L subgroup in AEG cohort

**Figure S2.** The workflow of the study

**Table S1**. The association between immune infiltration subtypes and clinical indexes in TCGA AEG cohort (*N* = 201).

| TCGA | Total | immunity_L (*N*=108) | immunity_H (*N*=93) | P |
| --- | --- | --- | --- | --- |
| Age |  |  |  | 0.487 |
| ≤65 | 95 | 54 | 41 |  |
| ＞65 | 106 | 54 | 52 |  |
| Gender |  |  |  | **0.016^a^** |
| Female | 50 | 19 | 31 |  |
| Male | 151 | 89 | 62 |  |
| TNM stage |  |  |  | 0.855 |
| Stage Ⅰ-Ⅱ | 87 | 47 | 40 |  |
| Stage Ⅲ-Ⅳ | 83 | 46 | 37 |  |
| NA | 31 | 15 | 16 |  |
| Site |  |  |  | **0.010^a^** |
| Cardia, NOS | 87 | 44 | 43 |  |
| Fundus of stomach | 40 | 15 | 25 |  |
| Lower third of esophagus | 74 | 49 | 25 |  |
| Grade |  |  |  | **0.001^a^** |
| G1-G2 | 50 | 32 | 18 |  |
| G3 | 75 | 26 | 49 |  |
| NA | 76 | 50 | 26 |  |

^a^ Indicate statistically significant (chi-square test, *P* < 0.05)

**Table S2.** The results of gene sets enrichment analysis between two immune infiltration subtypes (*P* < 0.05).

The results are shown in excel.

**Table S3.** 1470 lncRNAs that were differentially expressed between two immune infiltration subtypes (FDR < 0.05 and | logFC | >1).

The results are shown in excel.

**Table S4.** Six prognostic risk signatures closely related to immune infiltration.

| LncRNA | Regression Coefficient | Hazard Ratio(95%CI) | P |
| --- | --- | --- | --- |
| LINC01502 | -0.2656 | 0.7667(0.6002-0.9796) | 0.0336 |
| FLJ38122 | 0.4971 | 1.6439(1.2722-2.1242) | 0.0001 |
| C15orf32 | 0.1952 | 1.2155(1.0546-1.4011) | 0.0071 |
| LINC00706 | -0.235 | 0.7906(0.6141-1.0178) | 0.0683 |
| LINC01348 | 0.3437 | 1.4101(1.1111-1.7896) | 0.0047 |
| BCAR4 | 0.4109 | 1.5081(1.2220-1.8612) | 0.0001 |

**Table S5.** Requested public datasets of AEG samples in this study.

The results are shown in excel.

**Table S6.** key resource table

| **REAGENT or RESOURCE** | **SOURCE** | **IDENTIFIER** |
| --- | --- | --- |
| **Deposited Data** |  |  |
| AEG expression data: TCGA cohort | Genomic Data Commons | <https://portal.gdc.cancer.gov/> |
| AEG clinical data: TCGA cohort | UCSC Xena | https://xenabrowser.net/datapages/?hub=https://gdc.xenahubs.net:443 |
| AEG somatic mutation data (VarScan2) | Genomic Data Commons | https://portal.gdc.cancer.gov/ |
| UC cohort | (Mariathasan S et al., 2018) | http://research-pub.gene.com/IMvigor210CoreBiologies |
| **Software and Algorithms** |  |  |
| Gene Set Enrichment Analysis (GSEA) | (Subramanian et al., 2005) | http://software.broadinstitute.org/gsea/index.jsp |
| R (4.0.0) | NA | https://www.r-project.org/ |
| ESTIMATE | NA | https://r-forge.r-project.org/projects/estimate/ |
| COSMIC V2 | (Alexandrov et al., 2014) | https://cancer.sanger.ac.uk/signatures/signatures_v2/ |
| pRRophetic | (Geeleher P et al., 2014) | http://genemed.uchicago.edu/,pgeeleher/pRRophetic |

**
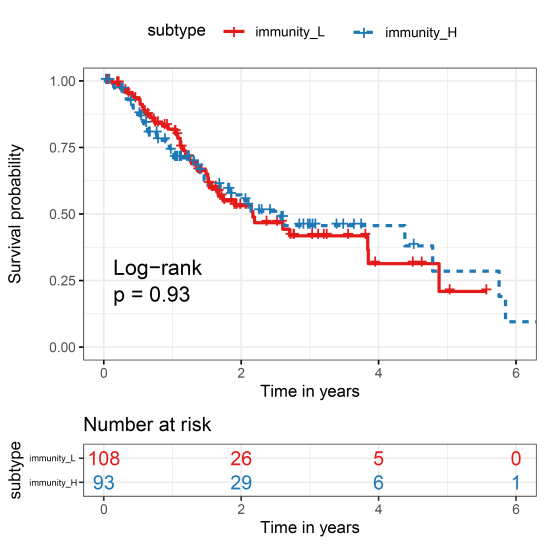
Figure S1.** Kaplan–Meier analysis of the immunity_H versus immunity_L subgroup in AEG cohort(*P =* 0.93).

**
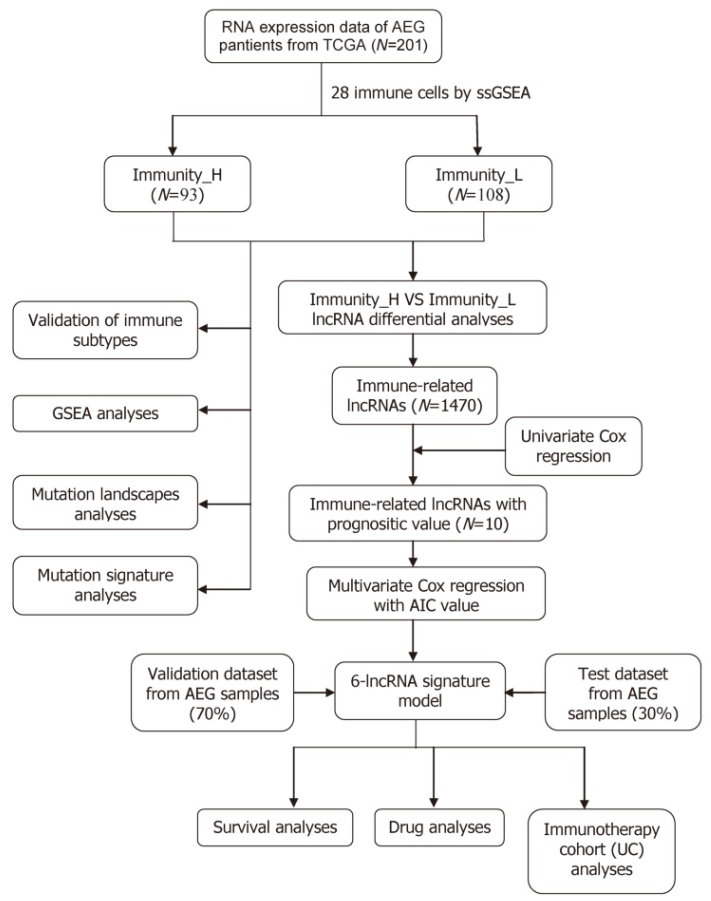
Figure S2.** The workflow of the study.
